# Changes in health-related quality of life during outpatient rehabilitation: a prospective observational cohort study in four patient groups

**DOI:** 10.1007/s11136-026-04167-2

**Published:** 2026-02-01

**Authors:** Daniëlla M. Oosterveer, Maud E. H. Ophelders, Bianca M. P. Mourits, Eline W. M. Scholten, Johanna M. A. Visser-Meily, Joris A de Graaf

**Affiliations:** 1 Basalt, Leiden, The Netherlands; 2 Department of Rehabilitation Medicine, Alrijne Hospital, Leiden, The Netherlands; 3 Department of Rehabilitation, Physical Therapy Science and Sports, UMC Utrecht Brain Center, University Medical Center Utrecht, Utrecht, The Netherlands; 4 Center of Excellence for Rehabilitation Medicine, UMC Utrecht Brain Center, University Medical Center Utrecht, PO Box 85500, 3508GA Utrecht, The Netherlands

**Keywords:** Quality of life, Rehabilitation, Brain injuries, Chronic pain, Nervous system diseases

## Abstract

**Purpose:**

To evaluate changes in health-related quality of life (HRQoL) of different patient groups, as measured using the EuroQoL (EQ5D) during outpatient multidisciplinary rehabilitation.

**Methods:**

Patients with acquired brain injury, chronic pain, neurodegenerative diseases or oncological diagnoses, who received outpatient multidisciplinary rehabilitation, were included in a multi-center prospective observational cohort study. They completed the EQ5D, consisting of an index and a Visual Analogue Scale (VAS), at the start of outpatient rehabilitation (T0) and 6 months thereafter (T1), and two perceived change questions (about quality of life and about general health) at T1.

**Results:**

Both EQ5D index and VAS improved for the total sample (*n* = 419, 68.8% females, mean age 54.5 years) and for each patient group, with the exception of the EQ5D VAS in patients with neurodegenerative diseases. The latter group showed less improvement, as measured on the EQ5D index, than patients with chronic pain (*p* = 0.004), and less on VAS compared to the other patient groups (all *p* < 0.05). At an individual level, 76.8% (304/396) of all patients reported improvement on the perceived change question about quality of life and 279/419 (66.6%) on the perceived change question about general health. Again, patients with neurodegenerative diseases had the lowest percentages (49/83 (59.0%) and 39/85 (47.0%), respectively).

**Conclusion:**

All patient groups improved on HRQoL during outpatient multidisciplinary rehabilitation, both at group and individual level. However, patients with neurodegenerative diseases showed slightly less improvement than other patient groups, which may reflect the progressive nature of their disease rather than lower rehabilitation effectiveness.

## Introduction

Rehabilitation has had different definitions over the years and has become more focused on well-being. Recently, Wade [1] proposed a definition in line with this focus: ‘Rehabilitation aids adaptation to changes associated with illness through accurate diagnosis and formulation, catalyzing adaptation, optimizing the environment and assisting the patient in making necessary changes’. Because of this focus on well-being, health-related quality of life (HRQoL, i.e. ‘how well a person functions in their life and his or her perceived well-being in physical, mental and social domains of health’) has become an important outcome in rehabilitation [2, 3]. One of the most widely used instruments to assess HRQoL is the EuroQoL-5 Dimensions (EQ5D), a generic patient-reported outcome measure (PROM) consisting of an index and a Visual Analogue Scale (VAS) with a low administrative burden; it is available in more than 170 languages [4].

Because rehabilitation acts on the adaptive process, improvements in HRQoL across different patient groups can be expected [1]. Indeed, previous studies in several patient groups have shown an effect of rehabilitation on HRQoL, measured with the EQ5D. Brands et al. [5] retrospectively studied 62 outpatients with acquired brain injury (ABI) and found a significant improvement on the EQ5D VAS. Similarly, a prospective study in 29 patients with chronic pain showed improvement in the EQ5D index and VAS one year after rehabilitation was started [6]. In a much larger cohort of 3233 patients with oncological diagnoses, the EQ5D index and VAS improved after inpatient rehabilitation [7]. However, in 214 patients with Multiple Sclerosis, a progressive disease, the effect of inpatient rehabilitation on the EQ5D index and VAS was not significant, although the EQ5D VAS did improve compared to patients on the waiting list [8, 9].

However, due to differences in design and setting, changes in HRQoL during rehabilitation across different patient groups remain unclear. For inpatient rehabilitation, Garratt et al. [10] demonstrated an improvement in the EQ5D for all patient groups. For outpatient multidisciplinary rehabilitation however, as far as we are aware there is no head-to-head comparison of the impact of rehabilitation on the EQ5D between patient groups. One could hypothesize that, in line with the definition of Wade [1], HRQoL will improve in all patient groups during outpatient rehabilitation, because the ability to adapt is not diagnosis-specific. On the other hand, differences in diagnosis-specific problems and disease course could very well lead to differences in the impact of multidisciplinary rehabilitation on specific dimensions of the EQ5D.

Therefore, this prospective observational cohort study aimed: (1) to evaluate changes in HRQoL, measured with the EQ5D index, VAS and dimensions, during outpatient multidisciplinary rehabilitation in all patients and in four specific patient groups (i.e. ABI, chronic pain, neurodegenerative diseases and oncological diagnoses); (2) to assess whether the four groups differed in EQ5D change score; and (3) to evaluate the size of change in HRQoL during outpatient rehabilitation: at the group level, by calculating effect sizes based on the EQ5D and at the individual level, by determining the number of patients reporting improvement in QoL and general health using perceived change questions.

## Materials and methods

### Design and setting

This study was part of the Measurement of Outcomes of Rehabilitation in The Netherlands (MUREVAN) study. In this multi-center prospective observational cohort study, data were collected from patients who were receiving inpatient or outpatient multidisciplinary rehabilitation treatment at the participating nine rehabilitation centers and rehabilitation departments of five hospitals in The Netherlands. The rehabilitation was provided by a multidisciplinary team (i.e. this team could include a rehabilitation physician, an occupational therapist, a physiotherapist, a speech therapist, a social worker, a psychologist and/or other therapists). Rehabilitation was goal-oriented, based on the needs and preferences of the patient. Eligible patients were invited to participate in this study by their treating rehabilitation physician between March 2023 and October 2024 and completed PROMs at the start of their rehabilitation (T0) and six months thereafter (T1).

It was considered that this study did not fall within the scope of the Dutch Medical Research Involving Human Subjects Act; it was approved by the research board of the University Medical Center Utrecht (UMCU; reference number 212217652), and was conducted in compliance with the Dutch General Data Protection Regulation and the Declaration of Helsinki [11]. Reporting was in accordance with the Strengthening the Reporting of Observational studies in Epidemiology (STROBE) Guidelines [12].

### Patients

Patients were included in the MUREVAN study when they (1) were aged ≥ 18 years old; (2) had been diagnosed with ABI, chronic pain, a neurodegenerative disease, an oncological diagnosis, or spinal cord injury; (3) received inpatient or outpatient multidisciplinary rehabilitation treatment, that was expected to last ≥ four weeks; (4) were able to complete PROMs in Dutch, and (5) provided informed consent. Patients with rapidly progressive disorders (e.g. amyotrophic lateral sclerosis or aggressive malignant brain tumors) were excluded.

For the current analyses, we included patients from the MUREVAN study, who were receiving outpatient multidisciplinary rehabilitation treatment. These patients were diagnosed with ABI, chronic pain, a neurodegenerative disease, or an oncological diagnosis. In addition, patients had to have completed PROMs (i.e. EQ5D and perceived change questions) at T0 and T1.

### Sociodemographic and clinical characteristics

Age, sex, educational level, living situation and country of birth were self-reported by patients at T0. The treating rehabilitation physician reported the diagnosis and the rehabilitation setting (i.e. inpatient or outpatient) at T0.

### Health-related quality of life

At both T0 and T1, HRQoL was measured using the EQ5D 5-Level [13]. This 5-Level EQ5D was found to show similar or better measurement properties compared to the 3-Level version [14]. This PROM consists of five items, each reflecting a dimension relevant to HRQoL, i.e. mobility, self-care, usual activities, pain/discomfort and anxiety/depression. Each item of the 5-Level EQ5D is scored on a 5-point Likert scale. This leads to an EQ5D index with a score ranging from − 0.33 (a health status worse than death) to 1 (healthy). Next to the index, the EQ5D consists of a Visual Analogue Scale (VAS), on which patients rate their overall health on a scale ranging from 0 (worst imaginable health) to 100 (best imaginable health).

### Perceived change questions

At T1, perceived changes in quality of life and general health over time were assessed by two questions: (1) “How has your quality of life changed compared to when you started your rehabilitation?”; and (2) “How has your general health changed compared to when you started your rehabilitation?”. Both perceived change questions were scored on a 7-point Likert scale (from ‘much worse’ to ‘much improved’).

### Statistical analyses

All data analyses were performed in IBM SPSS version 30.0 (IBM Corp: Armonk, NY, USA 2024). Data are presented as numbers (*n*) with percentages (%), as means with standard deviation (SD) or as medians and interquartile range (IQR) depending on their nature and distribution. The assumption of normality was assessed using histograms and Shapiro–Wilk tests.

To assess possible bias, age, sex and EQ5D of included patients were compared with those of patients who were excluded because they did not complete the EQ5D or the perceived change questions at both T0 and T1, using an independent samples t-test and chi-square test.

To evaluate changes in HRQoL during outpatient rehabilitation, change scores (T1 − T0) of the EQ5D index and VAS were computed for the total group and for each of the four patient groups (i.e. ABI, chronic pain, neurodegenerative diseases, and oncological diagnoses). Paired t-tests or Wilcoxon signed-rank tests were used to assess whether these changes in the EQ5D were significant.

To evaluate the changes in the five dimensions of the EQ5D index during outpatient rehabilitation, shifts in response distribution were analyzed with Marginal Homogeneity tests for each dimension per patient group. To account for multiple testing, a Bonferroni correction was applied, resulting in a significance threshold of *p* < 0.01.

To evaluate the differences in changes in HRQoL during outpatient rehabilitation across patient groups, a repeated measures ANOVA was conducted with Time (T0, T1) as the within-subject factor and patient group as the between-subjects factor. The ANOVA model tested for: (1) a main effect of Time (to measure overall within-person change, i.e. to assess whether EQ5D changes over time for the total population), (2) a main effect of Group (to measure between-group differences, i.e. to assess whether average EQ5D scores differed between the four patient groups), and (3) a Time × Group interaction (to measure differential change over time, i.e. to assess whether the EQ5D change over time differed between the four patient groups). The repeated measures ANOVA was also performed with age and sex as covariates as a sensitivity analysis. Post-hoc comparison of EQ5D changes between the four groups was carried out using a univariate ANOVA with a Bonferroni correction.

To evaluate the size of the change in HRQoL during outpatient rehabilitation at the group level, effect sizes (i.e. mean EQ5D change (T1 − T0) divided by SD at T0) and standardized response mean (SRM, i.e. mean EQ5D change (T1 − T0) divided by SD (mean change T1 − T0)) were calculated for the total group and for each of the four patient groups. Effect sizes of 0.20 were considered to be small, those of 0.50 te be moderate and 0.80 as large [15].

To evaluate the number of patients who experienced improvement in QoL and general health during outpatient rehabilitation, the proportion of patients who completed the perceived change questions with a positive answer (i.e. ‘slightly improved’, ‘moderately improved’ and ‘much improved’) was calculated. To assess whether this proportion differed between the four patient groups, Chi-Square tests with Bonferroni correction were used.

## Results

**Fig. 1:**
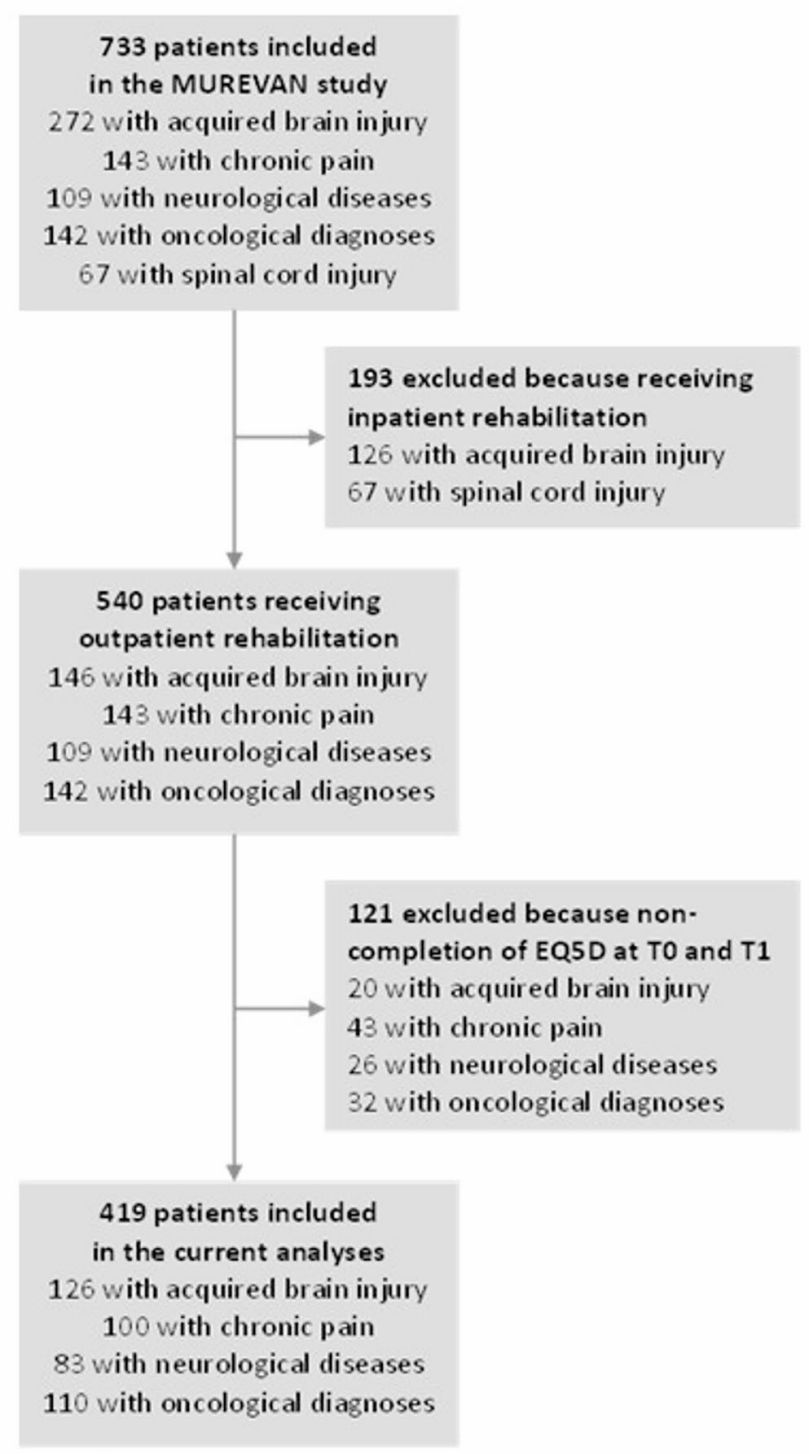
Flowchart of the study population

Between March 2023 and October 2024, 733 patients were included in the MUREVAN study. Of these patients, 540 received outpatient multidisciplinary rehabilitation: 146 patients with ABI, 143 with chronic pain, 109 with a neurodegenerative disease and 142 with an oncological diagnosis (Fig. 1). Of these 540 patients, 121 (22.4%) patients were excluded because they did not complete the assessment at T1. The remaining 419 included patients were older and had better EQ5D index and VAS than the 121 excluded patients, but the sex ratio of the two groups was not significantly different (mean age 54.5 (13.0) years versus 50.1 (14.6), *p* = 0.001, mean index 0.60 (0.19) versus 0.54 (0.21), *p* = 0.005, mean VAS 56.0 (17.1) versus 51.3 (17.6),* p* = 0.011, 68.8% females versus 63.6%, *p* = 0.574).

The sociodemographic characteristics of all included patients as well as those of the four patient groups are summarized in Table 1. Among the patients with ABI, stroke (69.0%) and traumatic brain injury (10.3%) were the most common diagnoses. Among the patients with neurodegenerative diseases, Multiple Sclerosis (43.4%) and neuromuscular diseases (41.0%) were the most common diagnoses.

**Table 1:** Sociodemographic characteristics of the included patients

	All diagnoses	Acquired brain injury	Chronic pain	Neuro-degenerative diseases	Oncological diseases
Number of patients	419	126 (30.1%)	100 (23.8%)	83 (19.8%)	110 (26.3%)
Mean age (SD) in years	54.5 (13.0)	58.0 (11.9)	49.2 (12.5)	57.3 (13.9)	54.5 (13.0)
Female sex	288 (68.8%)	64 (50.8%)	82 (82.0%)	48 (57.8%)	92 (83.6%)
High educational level	315 (75.2%)	84 (66.7%)	80 (80.0%)	62 (74.7%)	89 (80.9%)
Living alone	67 (16.0%)	18 (14.3%)	16 (16.0%)	15 (18.1%)	18 (16.4%)
Born in The Netherlands	392 (93.6%)	118 (93.7%)	92 (92.0%)	80 (96.4%)	102 (92.7%)

IQR, interquartile range; SD, standard deviation

### The change in HRQoL measured with the EQ5D during outpatient rehabilitation

Table 2 shows the mean EQ5D at T0 and T1 and the mean change scores for the total group and for each of the four patient groups. The EQ5D index ranged from 0.51 to 0.66 at T0 and from 0.64 to 0.77 at T1. Mean changes in the EQ5D index ranged from 0.06 to 0.15, and were significant in all patient groups. The EQ5D VAS ranged from 53.2 to 57.9 at T0 and from 59.0 to 70.9 at T1. Mean changes in the EQ5D VAS were not significant in patients with neurodegenerative diseases (1.9, *p* = 0.362), but did reach significance in the other patient groups (all *p* < 0.001).

**Table 2:** The impact of outpatient rehabilitation on health-related quality of life measured with the EQ5D in all patients and for the four patient groups

	All diagnoses *n* = 419	Acquired brain injury *n* = 126	Chronic pain *n* = 100	Neuro-degenerative diseases *n* = 83	Oncological diseases *n* = 110
*EQ5D index*					
Mean EQ5D index T0 (SD)	0.60 (0.18)	0.66 (0.18)	0.51 (0.20)	0.58 (0.20)	0.64 (0.14)
Mean EQ5D index T1 (SD)	0.71 (0.17)	0.77 (0.14)	0.66 (0.20)	0.64 (0.19)	0.73 (0.14)
Mean change EQ5D index (SD)	0.11 (0.17)	0.11 (0.15)	0.15 (0.21)	0.06 (0.15)	0.09 (0.16)
*p*-value*	< 0.001	< 0.001	< 0.001	< 0.001	< 0.001
Effect size	0.61	0.61	0.75	0.30	0.64
Standardized response mean	0.65	0.73	0.71	0.40	0.56
*EQ5D VAS*					
Mean EQ5D VAS T0 (SD)	56.0 (17.1)	57.9 (17.8)	53.2 (18.7)	57.2 (18.8)	55.3 (13.2)
Mean EQ5D VAS T1 (SD)	65.6 (17.6)	70.9 (15.5)	62.7 (19.8)	59.0 (19.9)	67.0 (13.3)
Mean change EQ5D VAS (SD)	9.6 (17.9)	12.9 (17.1)	9.6 (20.3)	1.9 (17.8)	11.7 (14.5)
*p*-value*	< 0.001	< 0.001	< 0.001	0.362	< 0.001
Effect size	0.56	0.73	0.51	0.10	0.88
Standardized response mean	0.54	0.74	0.47	0.11	0.81

SD, standard deviation. *p-values are the result of the paired t-test or Wilcoxon signed-rank tests

### The change in the dimensions of the EQ5D index during outpatient rehabilitation

**Fig. 2:**
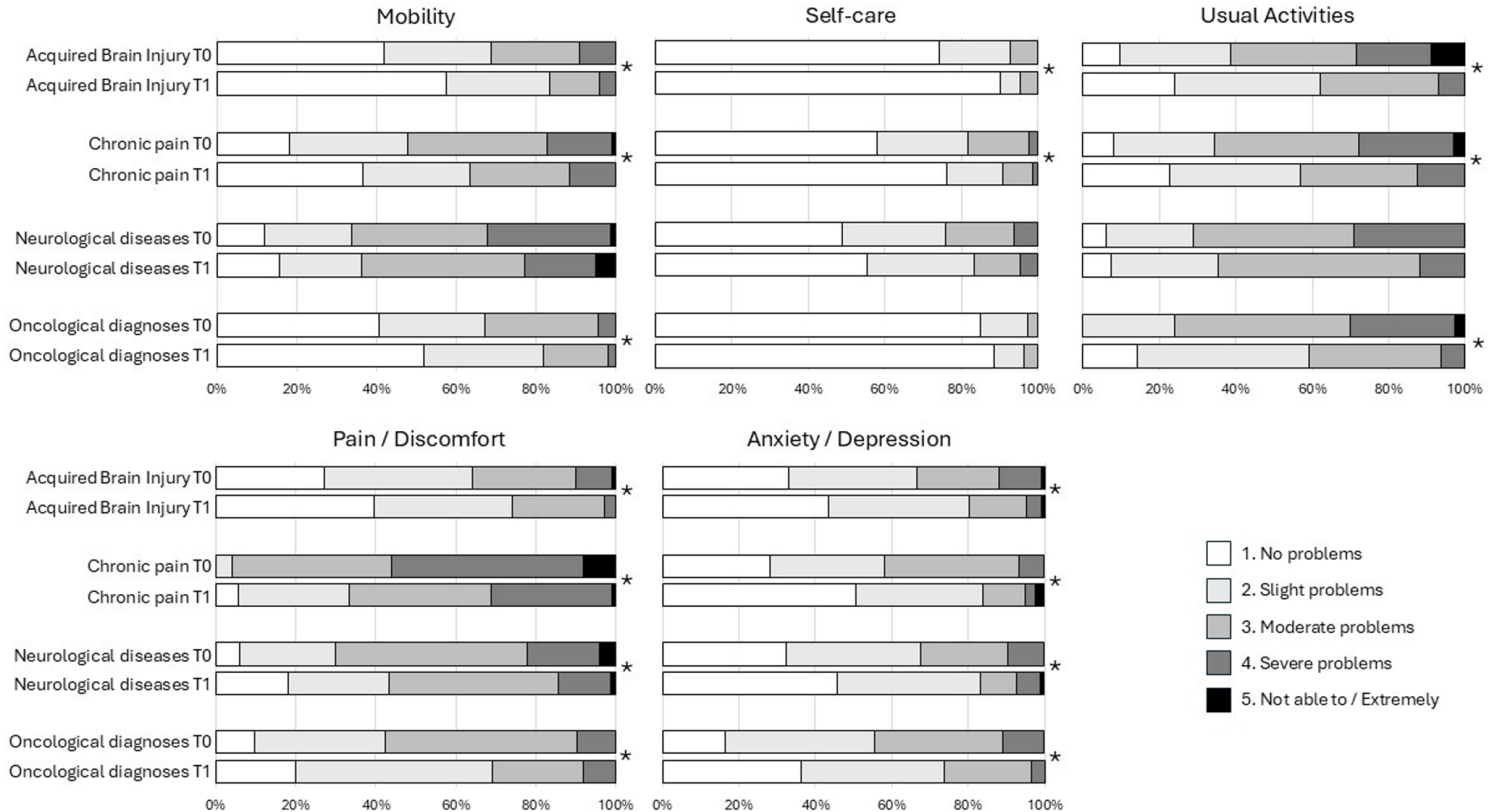
Distribution of responses on each dimension of the EQ5D index at T0 and T1 for the four patient groups. *Denotes a significant shift in response with a *p* < 0.01 based on a Marginal Homogeneity test

Figure 2 provides an overview of the responses on each dimension of the EQ5D index at T0 and T1. Shifts in response distribution were statistically significant for all dimensions in patients with ABI and chronic pain (*p* < 0.01). For patients with neurodegenerative diseases, there was no significant change in the dimensions mobility (*p* = 0.174), self-care (*p* = 0.058) or usual activities (*p* = 0.034). For patients with oncological diagnoses there was no significant change in the dimension self-care (*p* = 0.491).

### Differences in changes in HRQoL during outpatient rehabilitation across patient groups

Repeated measures ANOVA showed an overall change over time in EQ5D, for both the index and VAS for the total population (both *p* < 0.001) and showed that there was a difference in average EQ5D index and VAS between the four patient groups (both *p* < 0.001). Moreover, for both the index and VAS, the EQ5D change scores differed between the four patient groups (both *p* < 0.001). Correction for age and sex did not change the results. Post-hoc analyses of EQ5D change scores between the four groups demonstrated that the EQ5D index change score was lower in patients with neurodegenerative diseases compared to patients with chronic pain (*p* = 0.004), and that the EQ5D VAS change was lower in patients with neurodegenerative diseases compared to all other groups (ABI *p* < 0.001, chronic pain *p* = 0.017, and oncological diagnoses *p* < 0.001, respectively).

### The size of the change in HRQoL during outpatient rehabilitation at group level

Effect sizes and SRMs for the total group and for each of the four patient groups are shown in Table 2. Effect sizes for EQ5D index were small to moderate for patients with neurodegenerative diseases, and moderate to large for the other patient groups. For the EQ5D VAS, effect sizes were small for patients with neurodegenerative diseases, moderate for patients with chronic pain, moderate to large for patients with ABI and large for patients with oncological diagnoses.

### The number of patients who experienced improvement in QoL and general health during outpatient rehabilitation

The answers to the perceived change questions are shown in Table 3. In response to the question: “How has your quality of life changed compared to when you stared your rehabilitation?”, 76.8% of patients gave a positive answer, with a range between 59.0% to 85.5% for the patient groups. Responding to the question “How has your general health changed compared to when you started your rehabilitation?” 66.6% of patients gave a positive answer, with a range between 47.0 and 75.4% for the patient groups. For both perceived change questions, patients with a neurodegenerative disease scored lower than the other groups (*p* < 0.001).

**Table 3:** Answers on the anchor questions of all patients and of the four patient groups

	All diagnoses	Acquired brain injury	Chronic pain	Neuro-degenerative diseases	Oncological diseases
	*n* = 419	*n* = 126	*n* = 100	*n* = 83	*n* = 110
*How has your quality of life changed compared when you started your rehabilitation?*					
Much worse	1 (0.2%)	0 (0.0%)	0 (0.0%)	0 (0.0%)	1 (0.9%)
Moderately worse	10 (2.4%)	4 (3.2%)	1 (1.0%)	4 (4.8%)	1 (0.9%)
Slightly worse	20 (4.8%)	6 (4.8%)	2 (2.0%)	10 (12.0%)	2 (1.8%)
No change	61 (14.6%)	11 (8.7%)	18 (18.0%)	20 (24.1%)	12 (10.9%)
Slightly improved	116 (27.7%)	37 (29.4%)	21 (21.0%)	26 (31.3%)	32 (29.1%)
Moderately improved	123 (29.4%)	40 (31.7%)	31 (31.0%)	13 (15.7%)	39 (35.5%)
Much improved	88 (21.0%)	28 (22.2%)	27 (27.0%)	10 (12.0%)	23 (20.9%)
*How has your general health changed compared when you started your rehabilitation?*					
Much worse	2 (0.5%)	0 (0.0%)	0 (0.0%)	1 (1.2%)	1 (0.9%)
Moderately worse	11 (2.6%)	3 (2.4%)	1 (1.0%)	6 (7.2%)	1 (0.9%)
Slightly worse	27 (6.4%)	7 (5.6%)	6 (6.0%)	7 (8.4%)	7 (6.4%)
No change	100 (23.9%)	21 (16.7%)	26 (26.0%)	30 (36.1%)	23 (20.9%)
Slightly improved	105 (25.1%)	32 (25.4%)	30 (30.0%)	21 (25.3%)	22 (20.0%)
Moderately improved	107 (25.5%)	37 (29.4%)	17 (17.0%)	12 (14.5%)	41 (37.3%)
Much improved	67 (16.0%)	26 (20.6%)	20 (20.0%)	6 (7.2%)	15 (13.6%)

## Discussion

This study evaluated changes in HRQoL during outpatient multidisciplinary rehabilitation in different patient groups. Overall, both the EQ5D index and VAS improved significantly. Similar improvements were observed in the four patient groups, except for the EQ5D VAS in patients with neurodegenerative diseases. Repeated measures ANOVA demonstrated that changes in the EQ5D differed between patients groups: the improvement in EQ5D was smaller inpatients with neurodegenerative diseases. All five dimensions of the EQ5D index improved in patients with ABI and chronic pain, whereas patients with neurodegenerative diseases showed no significant changes in the dimensions mobility, self-care and usual activities. In patients with oncological diagnoses, only the self-care dimension did not improve. In line with these results, effect sizes for patients with neurodegenerative diseases were small to moderate, while those of the other groups were moderate to high. At the individual level, the majority of patients experienced improvements in QoL and general health. Patients with neurodegenerative diseases also reported improvements in QoL and general health, but less frequently than the other patient groups.

All patient groups in our study improved on EQ5D six months after starting outpatient rehabilitation, a result similar to those in patients receiving inpatient rehabilitation [10]. However, in this study by Garratt et al. [10], EQ5D change scores of a patient group were compared with all other groups and not between individual patient groups, which complicates comparison with our results. They reported less improvement in EQ5D index for patients with ABI, amputation, oncological diagnoses or fracture/trauma/degenerative diagnoses, and less improvement in the EQ5D VAS for patients with ABI, amputation or combined complex disorders compared to all other groups. These findings may partly be explained by relatively high baseline scores in their cohort. In our cohort, we also observed high baseline scores in patients with ABI (both the EQ5D index and VAS) and in patients with oncological diagnoses (the EQ5D index). As expected, more problems in the dimensions Mobility and Self-care the inpatients in the study of Garratt et al. [10] than in our outpatients, reflecting differences in the indication for inpatient versus outpatient rehabilitation.

Regarding the EQ5D scores, Brand et al. [5] reported similar EQ5D VAS scores (they did not report the EQ5D index) in their ABI population: a mean VAS of 59.9 (SD 17.4) at the start of rehabilitation and 70.4 (SD 12.4) posttreatment, compared to a mean VAS of 57.9 (SD 17.8) and 70.9 (SD 15.5), respectively in our patients with ABI. In contrast, the 24 patients with chronic pain studied by Hallstram et al. [6] had lower baseline scores (mean index of 0.14 (SD 0.31) and VAS of 36.3 (SD 19.2), respectively) and also lower scores after one year (mean index 0.35 (SD 0.38) and VAS 52.1 (SD 22.2), respectively). This suggests that their patients were more severely affected, possibly due to stricter inclusion criteria for rehabilitation [6]. They did not, however, clearly describe these inclusion criteria, whereas in our study, eligibility was based on the judgement of the treating rehabilitation physician.

Although Klocker et al. [7] studied the EQ5D 3-level version without computing an index in inpatient rather than outpatient oncological rehabilitation, their results were similar to ours. They reported that mean severity levels across the dimensions decreased, while the EQ5D VAS improved from 60.7 at start of rehabilitation to 72.3 six months thereafter. At both time points, most patients reported no problems with self-care, whereas improvements were observed in the other dimensions, consistent with our results.

At the individual level, changes in the EQ5D for specific patient groups have been scarcely reported. Only Boesen et al. [8] reported in patients with Multiple Sclerosis that 44.7% improved on the EQ5D index and 52.9% improved on the EQ5D VAS six months after starting inpatient rehabilitation. However, they did not clearly describe how improvement in EQ5D was defined. Nevertheless, between 40 and 60% of patients showed improvement, which is consistent with the findings in our study using perceived change questions. Short et al. [16] reported changes in EQ5D at the individual level in three other patient groups, namely patients receiving pulmonary rehabilitation, patients receiving group-based community exercise, and patients receiving physiotherapy for bone and joint care. In these groups, 38.4%, 52.5%, and 73.2% improved in EQ5D index, respectively, and 45.8%, 43.6% and 55.4% improved in VAS. These fluctuations across patient groups were also seen within our groups, suggesting that both diagnosis and therapy may impact variability in EQ5D changes.

It would be of interest to investigate whether outpatient multidisciplinary rehabilitation could be improved for patients with neurodegenerative diseases. However, the progressive nature of neurodegenerative diseases might explain why these patients appear to show less improvement on HRQoL during outpatient multidisciplinary rehabilitation than the other three patient groups. It could be that maintaining the same level of HRQoL is a positive outcome in these patients, while for patients with recent stroke or traumatic brain injury _ (ABI group) this would be a negative outcome, i.e. no recovery took place. On the other hand, a previous study in patients with Multiple Sclerosis showed that rehabilitation goals were met, regardless of whether the disease was stable or progressing [17]. Although the goals are based on the patients’ needs and preferences, not all goals necessarily translate into improved HRQoL and may be better captured with outcomes other than HRQoL. Outcomes such as satisfaction with one’s functioning and self-regulation, as important features of adaptation to changes associated with illness, may be particularly valuable for these patients [18]. Furthermore, HRQoL measures other than the short EQ5D might provide a more comprehensive assessment, although at the cost of increased administrative burden for patients.

### Strengths and limitations

The strengths of our study include its prospective observational multi-center design with over 400 patients, which enhances the generalizability of the results. Moreover, the analyses of the four distinct patient groups provide insight, not only into the overall effects of outpatient rehabilitation on HRQoL, but also into the specific dimensions of HRQoL that are most affected.

Our study also has limitations. Firstly, 22.4% of the patients did not complete the assessment at T1, even though assessments could be completed digitally or on paper, based on the preference of the patient, with two reminders. This loss to follow-up may affect representativeness. Based on the differences in the EQ5D index and VAS at T0 between included and excluded patients, a sample with a better HRQoL at T0 was included. Secondly, additional information on the severity of the health problem, such as modified Ranking Score (mRS) for stroke and Expanded Disability Status Scale (EDDS) for Multiple Sclerosis, would help to gain greater insight into our study population and the effects of rehabilitation on HRQoL. Also more details about the outpatient multidisciplinary rehabilitation, such as intensity, duration and content, would be of interest to explore whether the observed variability in HRQoL can be explained. However these details were unavailable. Some patients still received multidisciplinary rehabilitation at T1, and their HRQoL may therefore have improved further after T1. A longer follow-up would have been of value to capture this further improvement and to assess whether improvements were sustainable in the long-term.

Lastly, HRQoL is a broad and complex construct, with both reflective and formative elements [19]. Furthermore, different definitions of HRQoL and QoL exist [2]. Therefore, measuring HRQoL comprehensively with a single PROM, such as the EQ5D or perceived change questions, remains a challange. Other outcomes, such as functional dependence, satisfaction and self-regulation could complement HRQoL and provide a more complete evaluation of the benefits of outpatient multidisciplinary rehabilitation.

In conclusion, our study demonstrates that all studied patient groups improved in HRQoL six months after starting outpatient multidisciplinary rehabilitation, as measured with the EQ5D. This supports Wade’s expectation that rehabilitation, by acting on the adaptive process, should be effective across different patient groups [1]. Moreover, at the individual level, the majority of patients experienced improvements in QoL and general health during rehabilitation. Patients with neurodegenerative diseases, however, appeared to improve slightly less than the other patient groups, likely due to the progressive nature of their condition.

Adding severity indicators of diseases and more details about the outpatient multidisciplinary rehabilitation could provide greater insight into the observed variability of HRQoL outcomes in future research. Other outcomes, such as satisfaction with one’s functioning and self-regulation, could complement HRQoL outcomes. These insights could help clinicians tailor rehabilitation more effectively, especially for patients with neurodegenerative diseases.

## Data Availability

The dataset used and analysed during the current study is available from the corresponding author on reasonable request via the public data repository DataverseNL ( [https://doi.org/10.34894/F5MIGX](https:/doi.org/10.34894/F5MIGX) **).**.
